# Meta-Analysis of Placebo-Controlled Trials of Levosimendan in Acute Myocardial Infarction

**DOI:** 10.3390/jcdd8100129

**Published:** 2021-10-07

**Authors:** Gabriele Tumminello, Alberto Cereda, Lucia Barbieri, Giuseppe Biondi-Zoccai, Stefano Lucreziotti, Antonio Mafrici, Stefano Carugo

**Affiliations:** 1 Cardiology Division, ASST Santi Paolo e Carlo, Department of Health Sciences, University of Milan, 20142 Milan, Italy; tskcer@hotmail.it (A.C.); stefano.lucreziotti@asst-santipaolocarlo.it (S.L.); antonio.mafrici@asst-santipaolocarlo.it (A.M.); 2 Fondazione IRCCS Cà Granda Ospedale Maggiore Policlinico, Division of Cardiology, Department of Clinical Sciences and Community Health, University of Milan, 20122 Milan, Italy; lb.luciabarbieri@gmail.com (L.B.); stefano.carugo@unimi.it (S.C.); 3 Department of Medical-Surgical Sciences and Biotechnologies, Sapienza University of Rome, 04100 Latina, Italy; gbiondizoccai@gmail.com; 4 Mediterranea Cardiocentro, 80138 Napoli, Italy

**Keywords:** Levosimendan, acute heart failure, acute myocardial infarction

## Abstract

The treatment of acute myocardial infarction is early revascularization. Heart failure and cardiogenic shock may complicate acute myocardial infarction despite applying the best available strategy. Levosimendan is a relatively new drug to treat heart failure with a peculiar mechanism of action: calcium sensitization of myocardial fibres. Levosimendan has a direct inotropic effect but also pleiotropic effects; through the K+ATP channel’s opening, it also has a vasodilator effect which may participate concretely in the global effects of the drug. The focus of the literature is on the anti-heart failure and anti-cardiogenic shock properties of Levosimendan, but it may have effects also preventing the development of myocardial insufficiency in acute myocardial infarction. The aim of the meta-analysis is to evaluate the effect of Levosimendan on acute myocardial infarction in placebo-controlled trials. Based on the eight studies selected, we found a beneficial effect of Levosimendan on acute and long-term mortality of patients affected by acute myocardial infarction. With caution in interpreting the results of this meta-analysis, our data support the idea that Levosimendan may already have a role in the treatment of acute ischemic heart disease. Further studies specifically designed to investigate the early role in the treatment of ischemic heart failure are needed.

## 1. Introduction

The first line of treatment for patients affected by acute myocardial infarction (AMI) is the early revascularization with PCI [1]. Cardiogenic shock complicates approximately 5% of myocardial infarctions needing adjunctive support. The utility on the long-term survival of mechanical support such as intra-aortic balloon pump (IABP) [2] or ECMO [3] is debated but their use is not so rare as a “last chance” therapy for cardiogenic shock. The use of intravenous inotropic agents (amines and phosphodiesterase inhibitors) in the acute setting is well known to be related to a worse long term outcome [4,5,6]: the mechanism of action is related to a stable increase of intracellular concentration of free calcium improving contractility on one side but having, on the other side, deleterious effects such as increasing myocardial energy consumption, ischemia induction and may even produce myocardial necrosis, cardiotoxicity, and arrhythmias. Levosimendan is a relatively novel inotropic agent with a calcium-sensitizing myofilament effect and ATP-dependent potassium channel opening mechanism of action [7]. Levosimendan increases the myocyte contractility without an increase of oxygen consumption and an improvement of diastolic function [8]; the cardiac effect is corroborated by arterial vasodilatation with an increase of the oxygen demand/supply ratio [7] and a cardioprotective effect. Despite the potential beneficial effects in the acute phase of myocardial infarction, the use of Levosimendan in recent guidelines is currently limited to patients with heart failure and a severe reduction in cardiac output not responding to standard therapy, especially in patients under a beta-blocker effect [1]. The possible underuse of this promising and intriguing drug is the lack of evidence in this particular setting and the fact that the literature is constituted of small and variable studies. The majority of studies have been performed in patients who already developed heart failure and/or cardiogenic shock, losing the possibility to analyse the potential benefits of Levosimendan in the early phase of this acute disease. Focusing on a population of decompensated patients where support therapy is mandatory, the majority of studies have been performed versus dobutamine or other supportive treatment and not versus placebo. There is still a gap of knowledge regarding the use of Levosimendan in the context of acute ischemic heart disease. Many studies have focused on the role of this drug in the most advanced stages of acute heart failure rather than on the early intermediate stages of acute ischemic heart failure (SCAI A-B stages of cardiogenic shock [9]). The available studies are outdated, small in size and with heterogeneous end points, so the available evidence is limited. In spite of everything, the haemodynamic effects of the drug that reduces afterload, the beneficial pleiotropic effects on myocardial stunning and the improvement of microcirculatory indices make Levosimendan still an interesting therapeutic prospect in AMI.

Purpose of the study: The study aimed to analyse the available data of the use of Levosimendan in the acute setting of myocardial infarction

## 2. Materials and Methods

### 2.1. Design

This review was registered in the International Prospective Register of Systematic Reviews (PROSPERO) and was conducted and presented according to best practice recommendations, including the Preferred Reporting Items for Systematic Reviews and Meta-Analyses (PRISMA) reporting guidelines [10].

### 2.2. Search Strategy

An online search was conducted in PubMed, Embase, Google Scholar, and Scopus databases on 21 July 2021 to detect the studies to include in the meta-analysis by four trained investigators. No language restriction was enforced. In addition, we used backward snowballing (i.e., scanning of references of retrieved articles and pertinent reviews). The search strategy aimed to include any controlled study with Levosimendan administration in AMI in adult humans. As search terms, we included “Levosimendan AND acute myocardial infarction”, “Levosimendan AND STEMI”,” Levosimendan AND primary PCI”, “Levosimendan AND acute ischemic cardiomyopathy”.

### 2.3. Study Selection

Two authors (AC, GT) reviewed all the abstracts to identify potential eligible trials. If possible, full-text articles were reviewed to evaluate if they met the inclusion criteria. If the complete text was not available, the corresponding author has been contacted to get further material. The inclusion criteria were reports of placebo-controlled clinical trials in adult patients in the acute phase of myocardial infarction; only original research articles were included. The exclusion criteria were reports comparing Levosimendan to other drugs; case reports/series and review articles were excluded. The eligibility of the retrieved items was independently assessed by two authors and the disagreement was resolved by consensus.

### 2.4. Outcomes Definition

Based on the available outcomes of the included studies, the following end-points were assessed and extracted. Primary outcomes were acute mortality (within 14 days from event) and one-year mortality. Secondary outcomes were heart rate (HR), blood pressure (BP), cardiac index (CI), cardiac output (CO), wedge pressure (Wedge), systemic vascular resistance (SVR). The following adverse events were considered: hypotension, arrhythmias (atrial and ventricular arrhythmias of new onset) and new ischemic events.

### 2.5. Data Abstraction and Study Characteristics

Two investigators abstracted the data using a data-recording table design for this purpose. Data collected included: patient baseline characteristics, study design, sample size, clinical setting, clinical outcomes and haemodynamic findings.

### 2.6. Risk of Bias and Quality of Evidence Assessment

We used the GRADE criteria to assess the quality of evidence for each study. The studies were classified in very low, low, moderate, and high quality according to study design, risk of bias (assessed by using the Cochrane Risk of Bias (RoB)-2 Tool with the exclusion of a single registry found [10]), consistency, directness, precision, publication bias, effect, dose-response, confounders, and spurious effects (Table 1).

### 2.7. Statistical Analysis

**Figure A1:**
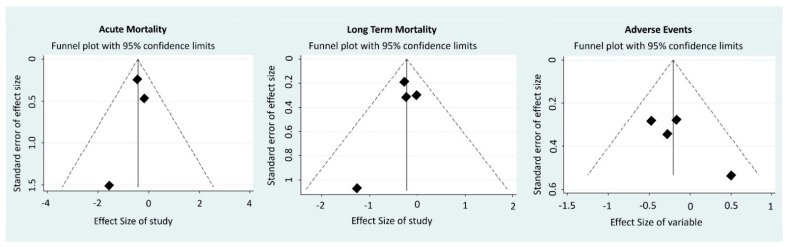
Funnel Plots.

Quantitative data are reported as mean and standard deviation where available. The categorical data are reported in percentages. The results are expressed in terms of odds ratios (OR) with 95% confidence intervals for dichotomous variables, or standardized mean difference (SMD) with 95% confidence intervals for continuous variables, with pooled estimates based on a random effect model. Statistical inconsistency was appraised by computing I^2^. Meta-regression was performed to explore the potential presence of effect modifiers. Funnel plots were visually inspected for the potential presence of small study effects, with regression tests to further corroborate the appraisal of publication bias (Appendix A Figure A1). Statistical significance was set at the 2-tailed 0.05 level. Computations were performed with OpenMetanalyst (Brown University, Providence, RI, USA).

## 3. Results

### 3.1. Subsection

#### 3.1.1. Included Studies

After careful examination of titles, abstracts, and full texts, 8 studies were eventually included in the meta-analysis [8,11,12,13,14,15,16,17]. The characteristics of each study are shown in Table 2. The quality of evidence assessment is shown in the last column of the table. The small sample size was a major limitation for most of the studies, and the only multicentre trial with an adequate sample size, RUSSLAN, divided the entire population into different subgroups assigned to different doses of Levosimendan, potentially limiting the power of the study. The characteristics of the sample population for each study are shown in Table 3. All the studies were balanced in terms of age of the population and prevalence of the female sex, as well as diabetes and previous myocardial infarction, with the population of the studies being composed mainly of STEMI patients. Notably, great variability was present in the timeline after the start of the treatment/placebo when haemodynamic parameters are collected in each study: from 120 h for Husebye to 20 min for Sonntag (Table 3).

#### 3.1.2. Meta-Analysis

**Figure 1:**
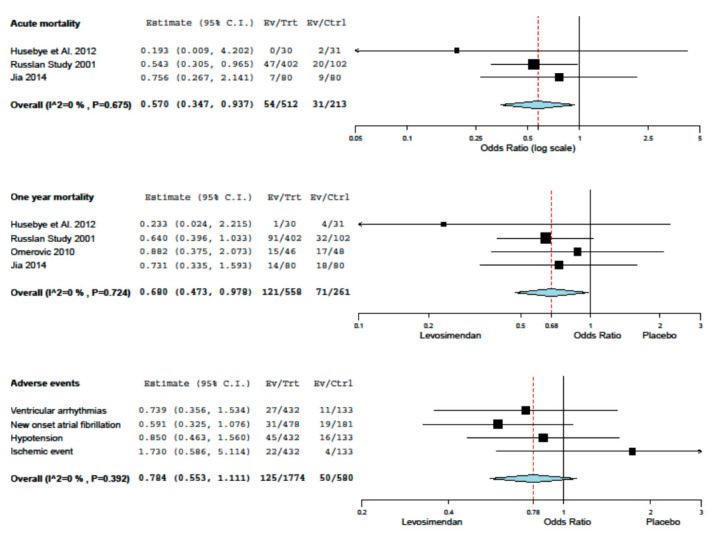
Meta-analisys.

Acute mortality of the eight studies included ranged from the lowest value of 3.2% (Husebye et al.) to the highest value of 13.2% (RUSSLAN). One-year mortality of the eight studies included ranged from the lowest value of 8.1% (Husebye et al.) to the highest value of 34.0% (Omerovic et al.). Levosimendan treatment was associated with a reduction in one year mortality and adverse event rates, without inconsistency (Figure 1). The results of the analysis focusing on haemodynamics and biohumoral parameters are summarized with respect to placebo in Table 4. A trend of the increase of cardiac index and reduction of wedge pressure was evident, with an inconsistent effect on blood pressure and heart rate. No evidence of small study effects was found upon the inspection of funnel plots or regression tests. Finally, possibly given the limited number of included studies, meta-regression analysis did not identify any major effect modifier.

## 4. Discussion

A clearly significant mortality reduction in the acute phase and long-term mortality induced by Levosimendan in AMI is evident. The metanalysis results are confirmed by the RUSSLAN study findings which is one of the main, bigger and better conducted studies on this topic. The treatment of AMI is well standardized and passes through the concept of an early revascularization of the culprit lesion [1], although several issues are still pending [18] and the evolution is not always benign. Incidence of heart failure after AMI is up to 36% [9] and it is the most powerful predictor of death [19]. Reducing the ischemic time is the key point to limit the myocardial damage [1] but adjunctive technical and pharmacological interventions may help to reach the goal [20]. In the acute ischemic field, the haemodynamic properties of Levosimendan may explain part of the benefits. Levosimendan, over an inotropic effect, has a positive effect on ventriculo–arterial coupling, peripheral vasodilation consequently increasing tissue perfusion, anti-stunning effects and anti-inflammatory effects [7]. These properties may explain the other part of the benefit, in particular not only treating an established condition of heart failure and/or cardiogenic shock but also preventing its development. If the pure haemodynamic effect of Levosimendan, as well as the IABP support, may act on very short mortality, it disappears after the stop of the infusion and with it, the effect on long term mortality, as well as the IABP support [21]. On the contrary, the protective effects on the myocyte of Levosimendan, which IABP support cannot have, may have had an impact on long-term mortality. The short-term and one-year mortality figures are pretty clear. Levosimendan, thanks to its non-haemodynamic pleiotropic effects, could exert a rescue action on the ischemic myocardium. Increasing the local vasodilation and ameliorating the coupling required/supply of oxygen Levosimendan may act favourably in the acute ischemic setting. The opening of K+ATP channels in the cardiac mitochondria induced by Levosimendan exerts direct cardioprotective effects [22]. In animal models. Levosimendan reduces myocardial infarct size and increases left ventricular function after acute coronary occlusion [23] through anti-ischemic [24], anti-remodelling [25] and anti-apoptotic effects [26]. Its action in humans and normal clinical practice may replicate the animal models preserving more myocardium and preventing reverse remodelling and therefore post-infarct heart failure in the first year of the acute event. Our findings suggest that the role of Levosimendan may go over the simple treatment of cardiogenic shock but may play a role in the treatment of high-risk AMI patients. To our knowledge, all the studies involving Levosimendan focus their effort on patients that have already manifested signs and/or symptoms of heart failure. The role of Levosimendan in preventing the development of heart failure after a significant ischemic insult is unknown. If nowadays the role of Levosimendan is limited by the lastest guidelines [1] only to a very restricted number of patients with cardiogenic shock, this has been led by a vast amount of literature which has concentrated its attention to a point on the timeline of the acute ischemic heart disease displaced too far from the acute event. If the efficacy of Levosimendan has been tested on a patient with an SCAI grade C-D [27], it is not excluded that it may have also a role in the earlier phases, SCAI grade A-B, preventing the progression of heart failure. Levosimendan appears to be well tolerated in this kind of population; nevertheless, all the studies considered have been performed on well-selected and highly monitored patients. Twenty years after its discovery, this drug still has underestimated therapeutic potential. Its applications overlap with those of short-term mechanical support (IABP, Impella, ECMO), strategies not so widely available in the past. Is it worthwhile to undertake new studies in this field? Dedicated trials will be needed to explore the protective significance of Levosimendan in acute ischemic heart disease capable of combining hard-endpoints (mortality, heart failure) with soft endpoints derived from imaging and microcirculation study.

## 5. Conclusions

The overall heterogeneity of the studies and the potential biases require caution in interpreting the results of this meta-analysis. Our data support the idea that Levosimendan may already have a role in the treatment of acute ischemic heart disease. Further studies, specifically designed to investigate the early role in the treatment of ischemic heart failure are needed.

## Figures and Tables

**Table 1:** Cochrane Risk of Bias (RoB)-2 Tool analysis.

Intention to Treat	Unique ID	Study ID	Experimental	Comparator	Outcome	Weight	D1	D2	D3	D4	D5	Overall
	1	Husebye	Levosimendan	Placebo	WMSI	1	**+**	**+**	**+**	**!**	**+**	**!**
	2	Wu X et al.	Levosimendan	Placebo	Mortality	1	**+**	**+**	**!**	**!**	**+**	**+**
	3	Sonntag et al.	Levosimendan	Placebo	Invasive Hemodynamics	1	**+**	**+**	**+**	**-**	**+**	**+**
	4	Moiseyev et al.	Levosimendan	Placebo	Mortality	1	**+**	**+**	**+**	**+**	**+**	**+**
	5	De Luca et al.	Levosimendan	Placebo	Hemodynamics	1	**!**	**!**	**+**	**+**	**!**	**!**
	6	De Luca et al.	Levosimendan	Placebo	Echocardiography Param.	1	**+**	**!**	**+**	**+**	**+**	**!**
	7	Jia Z et al.	Levosimendan	Placebo	Mortality	1	**+**	**!**	**+**	**+**	**+**	**!**
												
					**+**	Low risk	**D1**	**Randomisation Process**				
					**!**	Some concerns	**D2**	**Deviation from the intended interventions**				
					**-**	High risk	**D3**	**Missing outcome data**				
							**D4**	**Measurement of the outcome**				
							**D5**	**Selection of the reported result**				

**Table 2:** Characteristics of Studies Involved in Meta-Analysis.

Authors	Title. *Journal of Publication*	Sample Size (N)	Study Design	Variable-Outcome	Main Findings	Potential Bias	Quality of Evidence
Husebye T et al.	Levosimendan in acute heart failure following primary percutaneous coronary intervention-treated acute ST-elevation myocardial infarction. Results from the LEAF trial: a randomized, placebo-controlled study [11]	61	Single centre, double-blind placebo control trial,	Improvement in wall motion score index (WMSI)	Levosimendan treatment improved contractility in post-ischaemic myocardium in patients with PCI-treated STEMI complicated by HF. The treatment was well tolerated, without any increase in arrhythmias.	Small sample size, long enrolment, use of WMSI	Moderate
Wu X et al.	Enhancement of myocardial function and reduction of injury with levosimendan after percutaneous coronary intervention for acute myocardial infarction: a pilot study [12]	30	Single centre, randomized 2:1 placebo control, open-label trial,	Haemodynamic and echocardiographic evaluations	Levosimendan can significantly improve the myocardium function of patients with myocardial stunning after PCI.	Small sample size, haemodynamic evaluation only at 48 h from the event	Moderate/ High
Sonntag S et al.	The calcium sensitizer levosimendan improves the function of stunned myocardium after percutaneous transluminal coronary angioplasty in acute myocardial ischemia [13]	24	Single centre, double-blind placebo control trial,	Invasive haemodynamic evaluation	Levosimendan improved the function of stunned myocardium without obvious impairment of diastolic function.	Small sample size, haemodynamic evaluation only at 20 min from the event	Moderate/ High
Moiseyev vs. et al.	Safety and efficacy of a novel calcium sensitizer, levosimendan, in patients with left ventricular failure due to an acute myocardial infarction. A randomized, placebo-controlled, double-blind study (RUSSLAN) [14]	504	Multicenter, double-blind placebo control trial	Primary endpoints: hypotension or myocardial infarction. Secondary endpoints: overall death, heart failure	Levosimendan at doses 0.1–0.2 microg × kg(^−1^) × min(^−1^) did not induce hypotension or ischaemia and reduced the risk of worsening heart failure and death in patients with left ventricular failure complicating acute myocardial infarction.	Mortality outcome was not primary endpoint, different doses of Levosimendan tested	Moderate/ High
Omerovic E et al.	Levosimendan neither improves nor worsens mortality in patients with cardiogenic shock due to ST-elevation myocardial infarction [15]	94	Multicenter registries, prospective cohort of the SCAAR registry vs. prospective cohort of the RIKS-HIA registry	1 month and 1 year mortality	The use of levosimendan neither improves nor worsens mortality in patients with CS due to STEMI. Well-designed randomized clinical trials are needed to define the role of inotropic therapy in the treatment of CS.	Comparison between two population in different periods of time and therapeutical indications	Low/ Moderate
De Luca et al.	Levosimendan improves haemodynamic and coronary flow reserve after percutaneous coronary intervention in patients with acute myocardial infarction and left ventricular dysfunction [16]	26	Single centre, open label placebo control trial	Haemodynamic parameters, invasive evaluation of coronary flow reserve	Levosimendan, given intravenously after a PCI procedure in patients with AMI and LV dysfunction, significantly improves haemodynamics and CFR, compared with placebo.	Small sample size, evaluation only at 10 min from the start of the infusion	Moderate
De Luca et al.	Effects of levosimendan on left ventricular diastolic function after primary angioplasty for acute anterior myocardial infarction: a Doppler echocardiographic study [8]	52	Single centre, open label placebo control trial	Echocardiographic evaluation	Levosimendan, after primary angioplasty in patients with anterior acute myocardial infarction, appears to improve the Doppler echocardiographic parameters of left ventricular diastolic function.	evaluation at 24 h from the start of the infusion, diastolic function as the only end point	Moderate
Jia Z et al.	Efficacy of intravenous levosimendan in patients with heart failure complicated by acute myocardial infarction [17]	160	Single centre, single-blind placebo control trial	Composite outcome death, myocardial ischemia or worsening heart at 14 days and 6 month	Short-term intravenous infusion of levosimendan appears to be more effective than placebo for treating patients with heart failure complicated by AMI.	long enrolment, single-blind study	Moderate

**Table 3:** Population clinical characteristics of studies involved in meta-analysis.

*Clinical Characteristics*	Husebye	Wu	Sonntag	Russlan	Omerovic	DeLuca 2005	DeLuca 2006	Jia
Number of patients	61	30	24	504	94	26	52	160
Age (M)	63.9	63	60	67.2	66.0	57.2	61.3	63.1
Female sex (%)	30	10	29	48	26	34	25	40
Diabetes (%)	10	-	25	14.3	25.5	15.4	25	18.7
Previous AMI (%)	18	-	-	27	18	-	-	-
STEMI (%)	100	100	100	100	100	100	100	46,9
Anterior STEMI (%)	-	-	54	-	-	65.4	88.5	-
IABP (%)	28	-	-	-	77	-	-	11,2
Vasopressors/Inotropy (%)	8	-	100	7	76	-	-	-
Ejection Fraction (M)	41.4	-	-	-	-	32.8	38.4	29.2
Killip Class III/IV (%)	-	100	-	-	-	34.6	44.2	45.5
Acute Mortality (%)	3.2	-	-	13.2	-	-	-	10
Long Term Mortality (%)	8.1	-	-	24.4	34.0	-	-	20
Time of Infusion (h)	120	48	0,3	6	-	0,3	24	36

Abbreviations: AMI, acute myocardial infarction; STEMI, ST-elevation myocardial infarction; IABP: intra-aortic balloon pump.

**Table 4:** Qualitative haemodynamic effect of Levosimendan in studies involved in meta-analysis.

Studies	HR	BP	CI	Wedge	SVR	Tpn	CPK	BNP
**Husebye T et al.**	//	//	//	//	//	//	//	**=**
**Wu et al.**	**=**	**=**	**↑**	**↓**	**↓**	**↓**	**=**	**↓**
**Sonntag et al.**	**↓**	**↓**	**=**	**↓**	//	//	//	//
**RUSSLAN**	**=**	**=**	//	//	//	//	//	//
**Omerovic**	//	//	//	//	//	//	//	//
**De Luca 2005**	**=**	**=**	**↑**	**↓**	**↓**	//	//	//
**De Luca 2006**	**=**	**=**	//	//	//	//	//	//
**Jia**	//	//	//	//	//	//	//	//

Symbols: **↑** increase respect to baseline; **↓** decrease respect to baseline; **=** not significative variation respect to baseline; // data not present. Abbreviations: HR, heart rate; BP, blood pressure; CI, cardiac index; Wedge, wedge pressure; SVR, systemic vascular resistances; Tpn, Troponin; CPK, creatine phosphokinase; BNP, brain natriuretic peptide.

## Abbreviations

HR	Heart Rate
BP	Blood Pressure
CI	Cardiac Index
Wedge	Wedge Pressure
SVR	Systemic Vascular Resistance
Tpn	Troponine
CPK	Creatinine Phospho Kinase-MB
BNP	Brain Natriuretic Peptide
Compl	Complications Levosimendan vs. Placebo
